# Involvement of Epigenetic Modifications of GABAergic Interneurons in Basolateral Amygdala in Anxiety-like Phenotype of Prenatally Stressed Mice

**DOI:** 10.1093/ijnp/pyy006

**Published:** 2018-02-20

**Authors:** Chunting Zhu, Min Liang, Yingchun Li, Xuejiao Feng, Juan Hong, Rong Zhou

**Affiliations:** Department of Physiology, Nanjing Medical University, Jiangsu, China

**Keywords:** anxiety, DNMT1, epigenetic, GABAergic dysinhibition, GAD67, prenatal stress

## Abstract

**Background:**

Prenatal stress is considered a risk factor for anxiety disorder. Downregulation in the expression of GABAergic gene, that is, glutamic acid decarboxylase 67, associated with DNA methyltransferase overexpression in GABAergic neurons has been regarded as a characteristic component of anxiety disorder. Prenatal stress has an adverse effect on the development of the basolateral amygdala, which is a key region in anxiety regulation. The aim of this study is to analyze the possibility of epigenetic alterations of GABAergic neurons in the basolateral amygdala participating in prenatal stress-induced anxiety.

**Methods:**

Behavioral tests were used to explore the prenatal stress-induced anxiety behaviors of female adult mice. Real-time RT-PCR, western blot, chromatin immunoprecipitation, and electrophysiological analysis were employed to detect epigenetic changes of GABAergic system in the basolateral amygdala.

**Results:**

Prenatal stress mice developed an anxiety-like phenotype accompanied by a significant increase of DNA methyltransferase 1 and a reduced expression of glutamic acid decarboxylase 67 in the basolateral amygdala. Prenatal stress mice also showed the increased binding of DNA methyltransferase 1 and methyl CpG binding protein 2 to glutamic acid decarboxylase 67 promoter region. The decrease of glutamic acid decarboxylase 67 transcript was paralleled by an enrichment of 5-methylcytosine in glutamic acid decarboxylase 67 promoter regions. Electrophysiological study revealed the increase of postsynaptic neuronal excitability in the cortical-basolateral amygdala synaptic transmission of prenatal stress mice. 5-Aza-deoxycytidine treatment restored the increased synaptic transmission and anxiety-like behaviors in prenatal stress mice via improving GABAergic system.

**Conclusion:**

The above results suggest that DNA epigenetic modifications of GABAergic interneurons in the basolateral amygdala participate in the etiology of anxiety-like phenotype in prenatal stress mice.

**Significance Statement**

The present study provides new insight into investigating the mechanisms underlying the influence of prenatal stress (PRS) on adult behaviors. Since PRS mice shows a similar epigenetic signature to patients with anxiety disorder, PRS mice can be a valid animal model to study the epigenetic mechanisms underlying anxiety disorder and validate the novel antianxietic drugs. Furthermore, our study reveals the validity of DNA methyltransferase 1 (DNMT1) inhibitor in improving a variety of PRS-induced anxiety alterations. DNMT1 may represent a potential new molecular target to treat patients with anxiety disorder. Thus far, epigenetic drugs have been used in cancer treatment, because they often selectively reactivate tumor suppressor genes that are silenced by CpG island promoter methylation.

## Introduction

Brain development is an intricate and subtle process and greatly sensitive to stress and environmental factors. Clinical and preclinical studies have indicated that early-life stress including prenatal stress (PRS) has an adverse effect on the neurodevelopment leading to the permanent abnormality of brain structure and function. Anxiety disorder in childhood or adulthood is regarded as the common long-term outcome of the disrupted effect of early-life stress on brain development. For example, epidemiologic studies have indicated that children exposed to early adverse experiences are at increased risk for the development of depression and anxiety disorders in the adulthood (Heim and Nemeroff, 2001; Cartwright-Hatton, 2006). Consistently, animal studies have indicated that prenatally stressed rats or mice exhibit excessive anxiety-like behaviors in the elevated plus maze (EPM) and the open field test (OFT) (Vallee et al., 1997; Mairesse et al., 2007; Zuena et al., 2008; Papale et al., 2017) and an increased reactivity of hypothalamo-pituitary-adrenal (HPA) axis to stress indicative of a typical anxious phenotype that is observed in anxiety patients (Darnaudery and Maccari, 2008; Weinstock, 2008).

Abundant data have demonstrated that “persistency” is the most obvious characteristic of the effects of early-life stress. Although the mechanisms underlying the relationship between adverse developmental experiences and life-long phenotypic consequences are unclear, the epigenetic role of early-life stress in the pathogenesis of anxiety disorder was suggested in epidemiological studies (Murgatroyd et al., 2009; Meaney and Ferguson-Smith, 2010; Papale et al., 2017). An early-life stressor has been reported to increase the expression of DNA methyltransferase (DNMT1 and 3a) in the postnatal brain (Matrisciano et al., 2013; Dong et al., 2015). DNMT1 and DNMT3a are maintenance enzymes that selectively localize in GABAergic neurons, and the overexpression of these enzymes has been reported in GABAergic neurons of cortical or striatal brain areas in psychotic patients (Veldic et al., 2005; Veldic et al., 2007; Zhou et al., 2013). The promoter of glutamic acid decarboxylase (GAD) 67, which is one of GABAergic neuronal markers, has been proved to be embedded in large CpG islands and express methylation consensuses (Grayson et al., 2005; Costa et al., 2007). Decreased GAD67 expression and increased DNMTs expression, as well an increase in the methylation of GAD67 promoter, have recently been reported in the brain of adult mice prenatally exposed to stress (Matrisciano et al., 2012, 2013).

The GABAergic inhibitory circuit in the basolateral amygdala (BLA) has been identified as a key regulator of anxiety-like behaviors in both preclinical and clinical settings (Sanders and Shekhar, 1995; Allison and Pratt, 2003; Bueno et al., 2005; Ackermann et al., 2008). The deficit in GABAergic system in the BLA is implicated in the pathophysiology of neurodevelopmental psychiatric disorders (Quirk and Gehlert, 2003; Benes, 2010; Felix-Ortiz et al., 2013). Several studies have identified effects of PRS on the developing amygdala (Kraszpulski et al., 2006; Charil et al., 2010) and adult amygdala function (Weinstock, 2008; Sadler et al., 2011).

Considering that PRS has adverse effects on DNA methylation and the development of amygdala, we investigated whether PRS results in the increase of anxiety-related behaviors through an epigenetic GABAergic dysfunction in the BLA. The aim of the present study is to increase the understanding of epigenetic mechanisms underlying anxiety disorder by analyzing the interactions between genes and the environment in an animal model.

## Materials and Methods

The present studies were approved by the Animal Care and Use Committee of Nanjing Medical University. The protocols used here were in accordance with the guidelines published in the NIH Guide for the Care and Use of Laboratory Animals. All efforts were made to minimize the number of animals and their suffering.

### Animal Model Preparation

Pregnant C57BL/6J mice (Oriental Bio Service Inc) were individually housed and food and water ad libitum. Control dams were left undisturbed throughout gestation with a 12-h-light/-dark cycle (lights on at 7:00 am and off at 7:00 pm), whereas stressed dams were subjected to the restraint stress as described previously (Zheng et al., 2016) with slight modification. From the tenth day of pregnancy until delivery, the pregnant dams were restrained in a transparent tube (12 cm×3 cm) for 45 min 3 times/d and exposed to 24-h constant light. The day of birth was referred to as postnatal day 0 (PND 0). Weaning occurred at PND 21 to 22, after which the offspring of control and stressed dams (control and PRS mice) were group-housed by litter and sex. To minimize the effect of parent-to-offspring interaction per litter, one female offspring was randomly selected from each litter as object of study. Male offspring were not taken into experiments, because the anxiogenic effects of PRS were mainly observed in females but not males (Papale et al., 2017). Then, the following experiments were performed at PNDs 60 to 70.

### Behavior Analysis

To avoid the influence of the fluctuation of gonadal hormones in estrous cycle on anxious behaviors (Koonce et al., 2012), the behavioral experiments were performed at diestrus of female mice. According to the types of vaginal epithelium cells (leukocyte cells, nucleated cells, and cornified cells), diestrus, proestrus, and estrus were determined. Mice were used to examine anxiety-like behaviors on 3 mornings following OFT (on the first morning)→EPM (on the second morning)→light/dark box test (DLT on the third morning) sequence. The room was dimly lit during the tests. The detailed methods of the behavioral tests have been described previously (Chiba et al., 2012). Animals’ behaviors were videotaped and quantified via Ethovision 3.0 software (Noldus Information Technology Inc.).

#### OFT

The novel environment was a 35 cm×35 cm×25 cm white Plexiglas arena. Mice were placed in a corner and were allowed to freely explore the field for 10 min. Both total traveled distance and time spent in the center area were recorded.

#### EPM

The elevated plus maze consisted of 2 open and 2 closed arms with each arm projecting 50 cm from the center (a 10×10 cm area). The whole apparatus was placed 50 cm above the floor. Control and PRS mice were placed in the center of the EPM, facing a closed arm, and were allowed to freely explore the maze for 10 min. The number of 4-paw entries and the time spent in the 2 open arms were blindly analyzed.

#### DLT

The DLT test was performed in a rectangular box divided in 2 compartments (light and dark). A removable dark Plexiglas partition was used to divide the box into light and dark sides. Each animal was placed into the light side of the box, facing away from the dark side and allowed to explore both chambers of the apparatus for 10 min. The time spent in the light side and the number of entries into the dark compartment were scored.

### Hormones Assay

Two batches of mice were killed, one between 7:30 and 8:00 am and the other between 5:30 and 6:00 pm. Plasma was separated and stored at -20°C for hormones assay. The concentrations of plasma corticosterone and adrenocorticotropin (ACTH) were assayed by a ^125^I double-antibody radioimmunoassay kit (07-189105; MP Biomedicals) according to the instructions of the manufacturer. The ACTH assay has a sensitivity of 2 pg/mL and intra-assay and interassay CVs of <8%. The corticosterone assay has a sensitivity of 1 ng/mL and intra-assay and interassay CVs of <10%.

### Real-time RT-PCR

Mice were killed (2 h after lights on) and whole brains were extracted and immediately stored at -80°C until assayed. Brains sections (50 μm thick) were cut in the coronal plane using a cryostat. The BLA subfield was dissected from the frozen slices on dry ice. Total RNA was extracted using TRIzol (15596–026; Invitrogen) following the manufacturer’s instructions. Possible contamination with genenomic DNA was removed by an on-column DNase I (15200, Qiagen) treatment. mRNA was reverse transcribed using the high-capacity cDNA Reverse Transcription kit (4368814; Applied Biosystems) following the manufacturer’s instructions. The primer sequences of DNMT1, DNMT3a, DNMT3b, methyl CpG binding protein 2 (MeCP2), ten-eleven translocation 1 (TET1), GAD67, and GAPDH are shown in Table 1. These primer sequences were decided upon according to the previous reports (La Salle et al., 2004; Matrisciano et al., 2013; Dong et al., 2015). RT-PCR was performed using a LightCycler FastStart DNA Master SYBR Green I kit (3515869001; Roche) and an ABI Prism 7300 Sequence Detection System (Applied Biosystems). To improve the accuracy of the real-time PCR for quantification, amplifications were performed intriplicate for each RNA sample. The relative expression of genes was determined using the 2^-ΔΔCt^ method with normalization to GAPDH expression.

**Table 1.** Primers for the Reference and Target Genes

Gene	Forward Primer (5’ to 3’)	Reverse Primer (5’ to 3’)
DNMT1	TGACAGTGGTGCTGAAGAAGCCAT	AGAATGGAGCCTCGAATTCTGAGA
DNMT3a	AACAACAACTGCTGCAGGTGCTTT	ACTCCTGGATATGCTTCTGTGTGA
DNMT3b	TTCAGTGACCAGTCCTCAGACACGAA	TCAGAAGGCTGGAGACCTCCCTCTT
MeCP2	GGTTGTCTCCACTGCTACTTAC	GCTAACTTGGGTGCTGATCT
TET1	ACGCTGGAACAAGTGGTAGCCATA	TGAACGTTTGGGTCTTGGAGGTCT
GAD67	CTTTGGAGCTCTTCCTGATTGA	TTGTTTGAGGGCTGTCTCTG
GAPDH	ACCAGGGAGGGCTGCAGTCC	TCAGTTCGGAGCCCACACGC
CpG-rich GAD67 promoter	GAGGAGAGCGGGCCAAGA	GTGCCGCTCCACACGCC

### Western-Blot Analysis

For protein quantification, we conducted measurements as described in detail elsewhere (Matrisciano et al., 2012). The BLA was dissected on ice and the protein was extracted using a total protein extraction kit (KGP250KGP2100; KeyGEN Biotech). Anti-DNMT1 monoclonal antibodies (NB100-264; Imagenex) and anti-GAD67 polyclonal antibody (MAB5406; Millipore) were used to detect DNMT1 and GAD67 protein. Anti-β-actin monoclonal antibody (04-1116; Millipore) was used an internal antibody. The IMAGEQUANT analysis software was used to perform the densitometric analysis of interest bands. The values were presented as an optical density ratio with respect to β-actin.

### Chromatin Immunoprecipitation Assays

Chromatin immunoprecipitation (ChIP) assays were used to detect DNMT1 or MeCP2 binding to CpG-rich regions of GAD67 promoter. The Chip procedure was carried out using a ChIP kit (17–295; Upstate). Briefly, approximate 10 mg of the BLA was used for this procedure. Tissue was incubated at 37°C for 10 min with 500 mL of PBS containing 1% formaldehyde and a cocktail of protease inhibitors (P8340; Sigma). Tissue was homogenized in 300 mL of SDS lysis buffer (supplied by ChIP kit, Upstate), and the lysate was sonicated for 15 min on ice. Immunoprecipitation was performed overnight at 4 °C by the addition of 10 μg of ChIP grade DNMT1 (ab87656; Abcam) or MeCP2 (ab2828; Upstate) to the sonicated solution. An aliquot of the sonicated lysate without antibody was used as input to quantify the total amount of DNA in sample extracts. Protein-free DNA was extracted and used for detection and quantification of CpG-rich regions of GAD67 promoter by quantitative PCR. The primers of CpG-rich GAD67 promoter were decided by the reports of Matrisciano et al., (2013) and shown in Table 1. The level of immunoprecipitated GAD67 promoter by the DNMT1 or MeCP2 antibody was expressed as a percentage of the input DNA using the following equation: % (DNA-IP/total input)=2 [(Ct(10%input) – 3.32) – Ct (DNA-IP)]× 100%.

### Methylated/Hydroxymethylated DNA Immunoprecipitation

Methylated (5MC) and hydroxymethylated DNA (5HMC) on GAD67 promoter were assessed using MeDIP (C02010011; Diagenode) and hMeDIP kits (C02010034; Diagenode) followed by quantitative PCR. The procedures for sample treatment and immunoprecipitation are described in the kit instruction manuals. The following quantitative PCR, the primers of GAD67 promoter, and the calculation of the level of immunoprecipitated GAD67 promoter by the 5MC or 5HMC antibody were the same as those in ChIP assays.

### Electrophysiological Analysis

#### Slice Preparation

Mice were killed by decapitation and prepared for slices electrophysiology as described previously (Zhou et al., 2011). Their brains were quickly removed and placed in ice-cold oxygenated artificial cerebrospinal fluid (ACSF) consisting of (in mM) 124 NaCl, 2 CaCl_2_, 4.5 KCl, 1.0 MgCl_2_, 26 NaHCO_3_, 1.2 NaH_2_PO_4_, and 10 D-glucose and adjusted to pH 7.4 by bubbling with 95% O_2_/5% CO_2_ mixture. Coronal brain slices (400 μm thick) were cut using a vibrating microtome (Microslicer DTK 1500) in ice-cold oxygenated (95% O_2_/5% CO_2_) ACSF. The slices containing the BLA and external capsule (EC) were stored for a minimum of 1 h in oxygenated ACSF maintained at RT.

#### Field Potential Recording

For recording, the BLA slices were transferred to a chamber continuously perfused with oxygenated ACSF (2 mL/min) maintained at 30°C. Biphasic square wave pulses were applied at EC through a stainless-steel stimulation electrode (Figure 6A). Stimulation-evoked population spikes (PS) were recorded from the BLA by a glass micropipettes filled with 2 M NaCl (4–5 MΩ) connected to an Axoclamp2B amplifier (Axon Instruments). PS response was sampled using pCLAMP software (Axon Instruments). The PS amplitude was defined as the mean amplitude of the peak negativity, measured from the peak of the early and the late negativity. Paired-pulse inhibition (PPI) of the PS was evoked by double-pulse stimulation to EC at 50% of maximal stimulus strength and expressed as the ratio of the second PS amplitude to the first one. The interval between double pulses (20, 50, 75, 100 ms) was adopted to fall within the time course of the intracellularly recorded IPSP attributed to activation of GABAergic interneurons (Rainnie et al., 1991).

### Drug Administration

**Figure 1.**
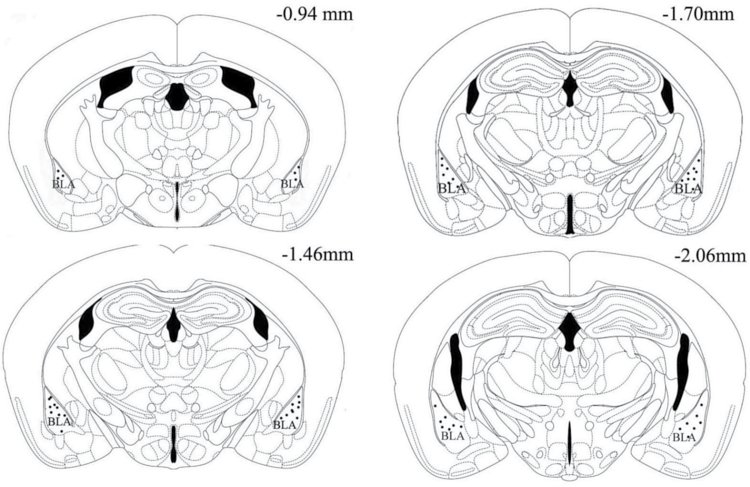
Illustration of the locations of the injection cannula tips in the basolateral amygdala (BLA) in all animals used in the study. Numbers show distances from bregma.

Cannulae were implanted with bilateral 27-gauge stainless steel cannulas into the bilateral BLA (Figure 1) according to the mouse brain in stereotaxic coordinates (Paxinos and Franklin, 2001). After a 7-day recovery period, animals received bilateral injection of the drugs with 0.3 μL/side through the infusion cannula (Nasehi et al., 2017). 5-Aza-deoxycytidine (5-aza-CdR; A3656; Sigma-Aldrich) was dissolved in 0.8% acetate and diluted to a concentration of 200 ng/μL in saline. 5-aza-CdR or vehicle was delivered through the cannula once a day for 7 consecutive days before the experiments.

### Data Analysis/Statistics

Data were retrieved and processed with the Origin 6.1 software (Micro-Cal Software Inc.). The group data were expressed as the means ±SEM. Two-tailed Student’s *t* test was used for comparisons between 2 groups. ANOVAs followed by Fisher’s protected least significant difference (PLSD) posthoc test was employed if more than 2 groups were compared. Statistical analysis was performed using Stata 7 software (Stata Corp). *P<*.05 was considered statistically significant. For statistical purposes, only one slice was studied per mouse in electrophysiological analysis.

## RESULTS

### PRS Mice Express the Increase of Anxiety-like Behaviors in Behavioral Experiments

#### OFT

**Figure 2.**
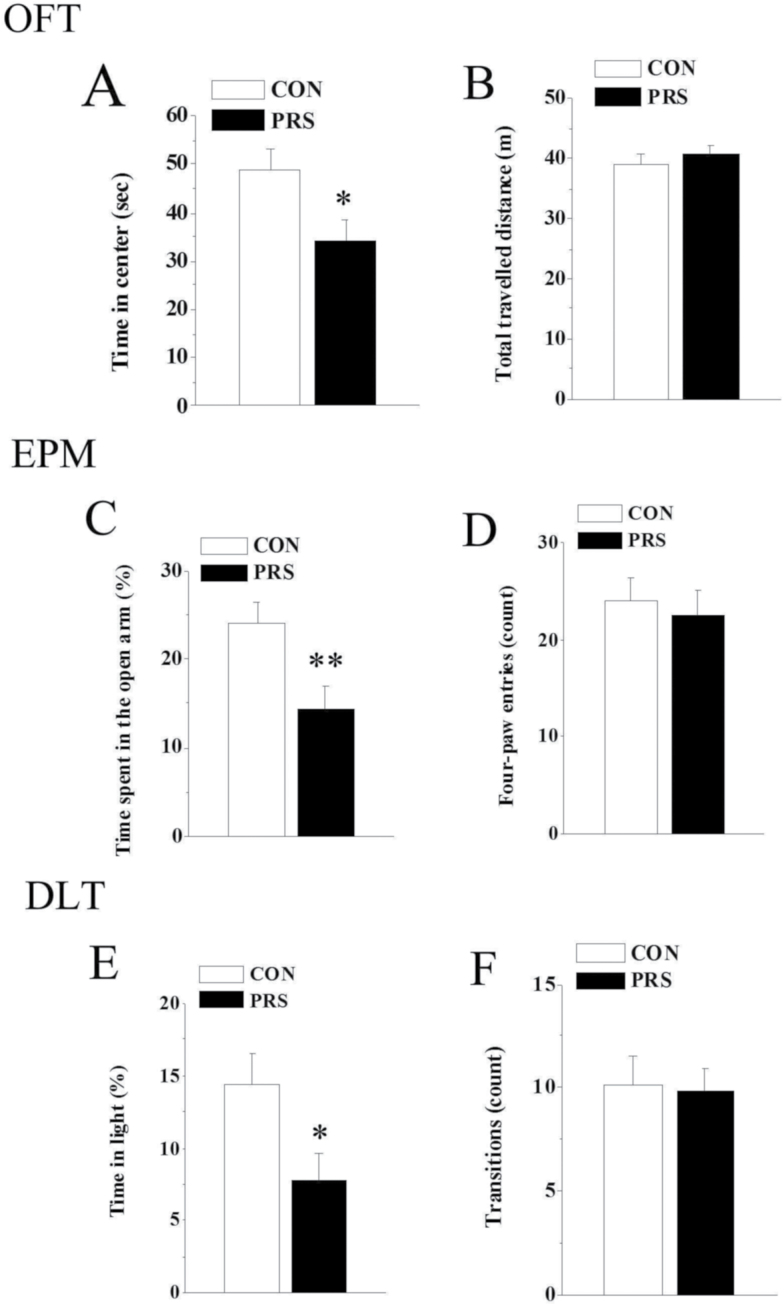
Prenatal stress (PRS) mice exhibit the increased anxiety-like behaviors. CON and PRS represent control and PRS mice, respectively. (A and B) In the open field test (OFT), PRS mice spent less time in the center area compared with control mice. (C and D) In the elevated plus maze (EPM), PRS mice showed a decreased percentage of time spent in the open arms compared with control mice. (E and F) In the light/dark box test (DLT), PRS mice spent less time in the light-box compared with control mice. All bars represent means±SEM; 2-tailed Student’s *t* test; *P<.05 and **P<.01 between the data of control (n=14) and PRS mice (n=14).

PRS mice showed a decrease in time spent in the central area of the open field in comparison with age-matched controls (t_(26)_=2.41, *P<*.05; Figure 2A). To exclude the influence of locomotion abnormality in this test, we also measured the locomotor activity of those mice. No significant difference in total traveled distance between control and PRS groups was observed in the present study (t_(26)_=0.27, *P* > 0.05; Figure 2B).

#### EPM

The percentage of time spent in the open arms was decreased in PRS offspring as compared with controls (t_(26)_=3.08, *P<*.01; Figure 2C). However, the number of 4-paw entries in the test area was not affected by PRS (t_(26)_=0.48, *P* > 0.05; Figure 2D).

#### DLT

PRS mice spent less time in the light-box of the DLT than controls (t_(26)_=2.26, *P<*.05; Figure 2E). No statistically significant differences were found in terms of the total number of transitions between the 2 groups (t_(26)_=0.146, *P* > 0.05; Figure 2F).

### PRS Results in the Potentiation of Circadian HPA-Axis Activity

**Figure 3.**
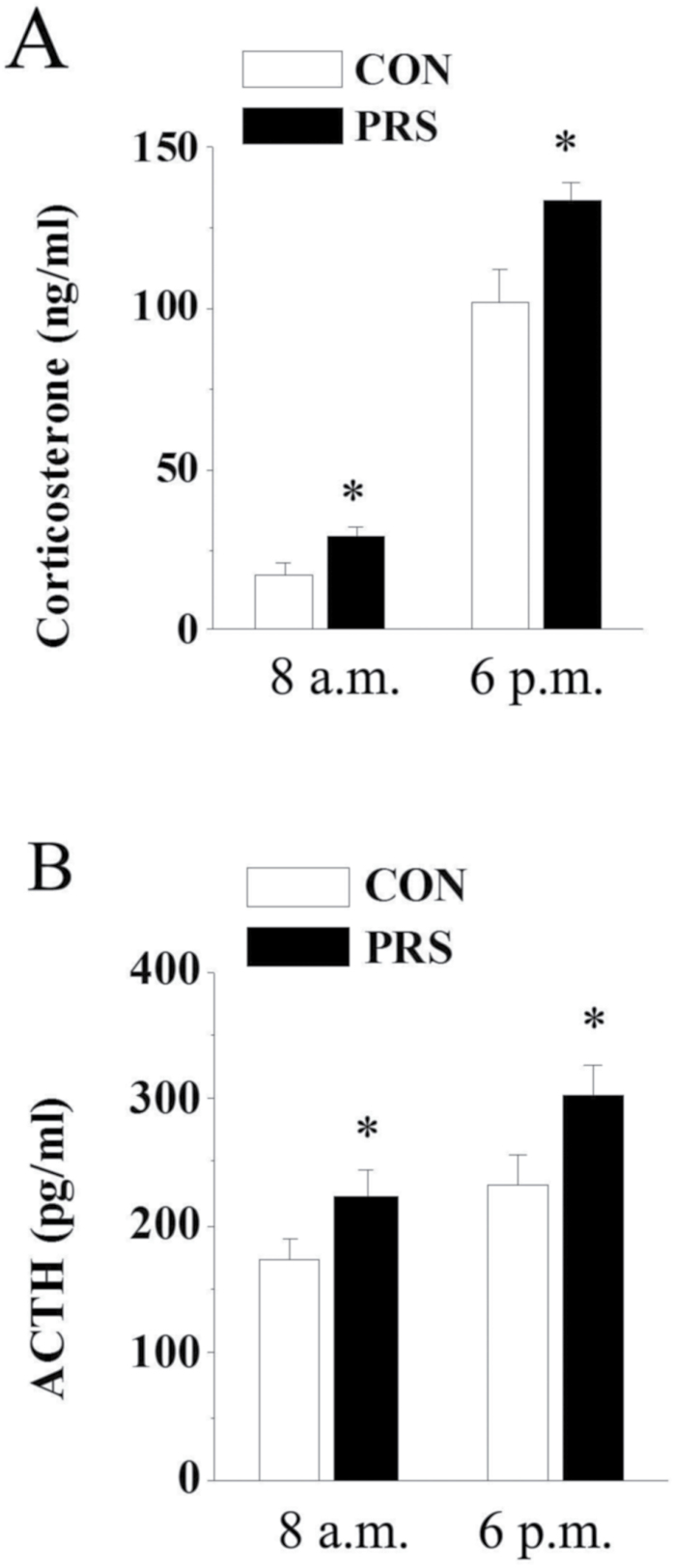
The basal activity of hypothalamo-pituitary-adrenal (HPA)-axis is potentiated in prenatal stress (PRS) mice. CON and PRS represent control and PRS mice, respectively. (A and B) PRS mice showed a significant increase in basal corticosterone (A) and adrenocorticotropin (ACTH) (B) concentrations. Sample collection was performed in the morning (8:00 am) and in the afternoon (6:00 pm). All bars represent means±SEM; 2-tailed Student’s *t* test; *P<.05 between the data of control (n=10) and PRS mice (n=12) at the same time.

To test whether PRS affects HPA-axis regulation leading to the change of anxiety-related behaviors, plasma was collected from control and PRS mice at circadian nadir (8:00 am) and peak (6:00 pm). As shown in Figure 3A–B, PRS mice exhibited a significant increase in both basal and peak corticosterone release compared with controls (basal: t_(20)_=2.51, *P<*.05; peak: t_(20)_=2.74, *P<*.05). Similarly, PRS mice had increased basal plasma ACTH and exhibited significantly increased peak ACTH (basal: t_(20)_=2.32, *P<*.05; peak: t_(20)_=2.21, *P<*.05).

### DNMT1 Excess Is Accompanied by the Decreased GAD67 Expression in the BLA of PRS Mice

**Figure 4.**
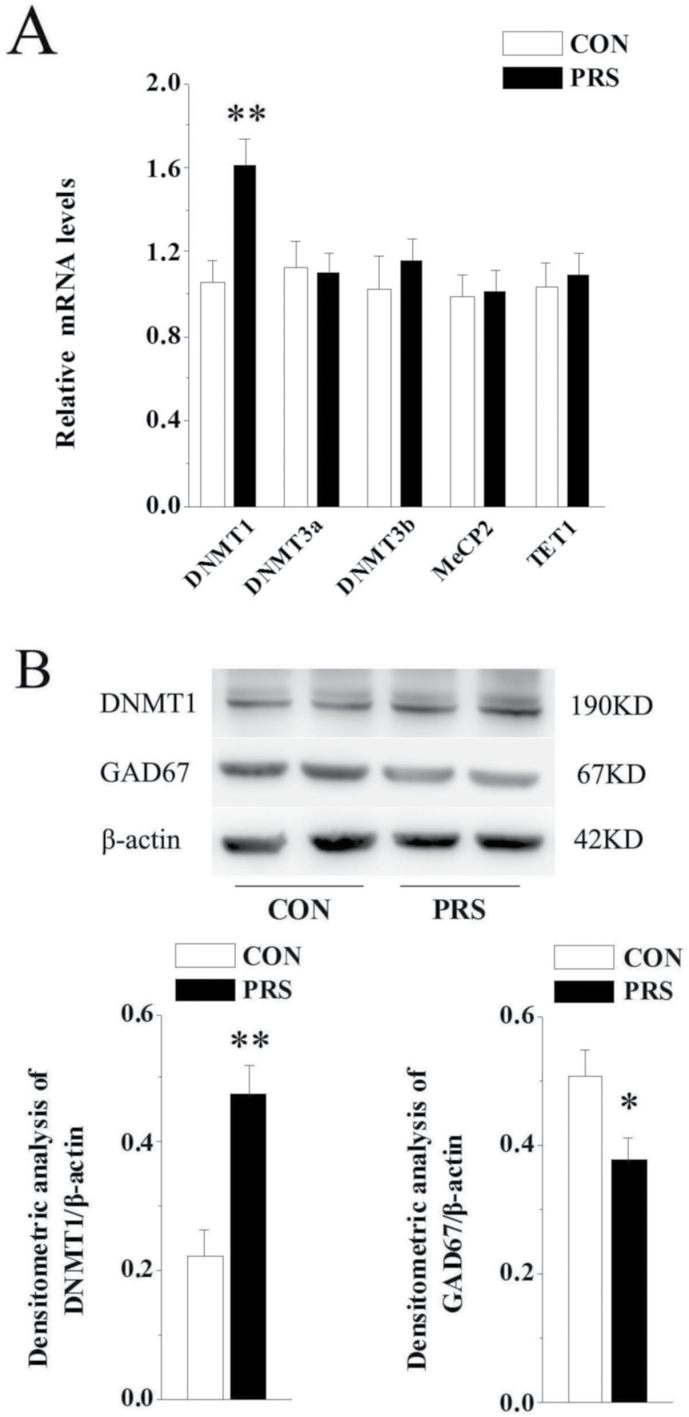
Elevated DNA methyltransferase 1 (DNMT1) levels are accompanied by the decrease of glutamic acid decarboxylase 67 (GAD67) expression in the basolateral amygdala (BLA) of prenatal stress (PRS) mice. CON and PRS represent control and PRS mice, respectively. (A) Quantitative RT-PCR results of DNMT1, DNMT3a, DNMT3b, MeCP2, and TET1 mRNA in the BLA of control and PRS mice. (B) Immunoblot data of DNMT1 and GAD67 normalized by β-actin protein levels. All bars represent means±SEM; 2-tailed Student’s *t* test; *P<.05 and **P<.01 between the data of control (n=8) and PRS mice (n=8).

Earlier reports suggest that an increase in DNMT levels is associated with a downregulation of GAD67 in postmortem brain tissue from patients with schizophrenia or bipolar disorders (Guidotti et al., 2011) and in brain of early postnatal stressed rats (Zhang et al., 2010). The results of real-time RT-PCR showed that in several detected enzymes associated with DNA methylation modification, only the expression of DNMT1 mRNA was significantly higher in the BLA of PRS mice compared with control mice (t_(14)_=3.16, *P<*.01; Figure 4A). The result of western blot indicated that associated with an increased level of DNMT1 (t_(14)_=4.29, *P<*.01), PRS mice showed a marked decrease in GAD67 protein level in the BLA (t_(14)_=2.41, *P<*.05; Figure 4B) compared with control mice.

### DNMT1-Induced Hypermethylation of GAD67 Promoter Results in the Downregulation of GAD67 in the BLA of PRS Mice

**Figure 5.**
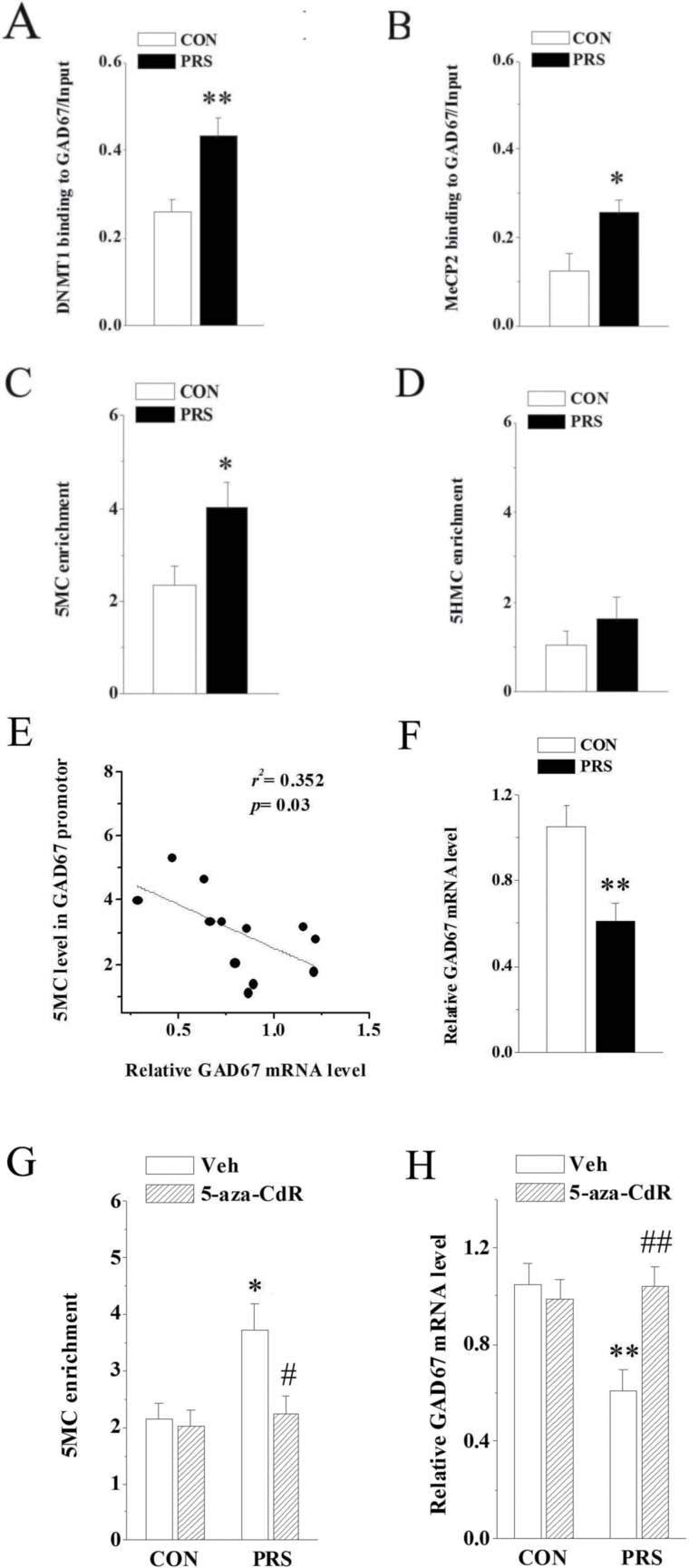
The hypermethylation of glutamic acid decarboxylase 67 (GAD67) promoter results in the downregulation of GAD67 in the basolateral amygdala (BLA) of prenatal stress (PRS) mice. CON and PRS represent control and PRS mice, respectively. (A and B) Data of DNA methyltransferase 1 (DNMT1) (A) or MeCP2 (B) binding to specific promoter regions of GAD67 in control and PRS groups. (C and D) The levels of 5MC (C) and 5HMC (D) on GAD67 promoter region in control and PRS groups. (E) The enrichment of 5MC on GAD67 promoter region is negatively correlated with the corresponding transcripts by Pearson correlation analysis. (F) Quantitative RT-PCR results of GAD67 mRNA in the BLA of control and PRS mice. (G and H) Effects of 5-aza-CdR on 5MC at GAD67 promoter and GAD67 transcript. All bars represent means±SEM; 2-tailed Student’s *t* test for A, B, C, D and F; 2-way ANOVA followed by protected least significant difference (PLSD) posthoc test for G and H; *P<.05 and **P<.01 between the data of control (n=6) and PRS mice (n=6) or between the data of vehicle-treated control (n=6) and vehicle-treated PRS mice (n=6); #*P<*.05 and ##*P<*.01 between the data of vehicle-treated PRS mice (n=6) and 5-aza-CdR-treated PRS mice (n=6).

To test whether the overexpression of DNMT1 in PRS mice correlates with an increased binding of DNMT1 to specific GAD67 CpG-rich promoter sequences, we measured the binding of DNMT1 to GAD67 promoter by ChIP assay. The results showed that the binding of DNMT1 to GAD67 promoter region was increased in PRS mice (t_(10)_=3.24, *P<*.01; Figure 5A). Moreover, the binding of MeCP2 to GAD67 was also significantly higher in PRS mice (t_(10)_=2.68, *P<*.05; Figure 5B) in the absence of the expression change of MeCP2 mRNA.

To investigate whether the decreased expression of GAD67 in PRS mice are the consequence of epigenetic modification on the corresponding DNA regulatory regions, we measured the enrichment of the 2 most important epigenetic marks: 5MC and 5HMC at the GAD67 promoter. As shown in Figure 5C, 5MC was enriched at GAD67 promoter in PRS mice compared with control mice (t_(10)_=2.66, *P<*.05). The 5HMC level in PRS mice was higher than control mice, but did not reach the significant level (t_(10)_=1.04, *P* > 0.05; Figure 5D). These findings suggest that PRS leads to CpG hypermethylation on GAD67 promoters. The enrichment of 5MC at GAD67 promoter was negatively correlated with the level of corresponding GAD67 transcript (*r*^*2*^=0.352, *P*=.03; Figure 5E), suggesting an epigenetic mechanism by which promoter methylation may be responsible for the downregulation of GAD67 in PRS mice (t_(10)_=3.83, *P<*.01; Figure 5F), and this possibility was therefore tested via detecting the effect of repeated intra-BLA injection of 5-aza-CdR, DNMT1 inhibitor on 5MC at GAD67 promoter, and GAD67 transcript. Two-way ANOVA displayed main effects of PRS and 5-aza-CdR treatment and their interaction on 5MC at GAD67 promoter (PRS: F_(1,24)_=6.28; *P<*.05; 5-aza-CdR: F_(1,24)_=4.89; *P<*.05; PRS×5-aza-CdR: F_(1,24)_=4.71; *P<*.05; Figure 5G) and GAD67 transcript (PRS: F_(1,24)_=5.77; *P<*.05; 5-aza-CdR: F_(1,24)_=5.01; *P<*.05; PRS×5-aza-CdR: F_(1,24)_=8.53; *P<*.01; Figure 5H). The treatment with 5-aza-CdR abolished both the alterations of 5MC level at GAD67 promoter (*P<*.05) and GAD67 transcript (*P<*.01) in PRS mice.

### DNMT1-Mediated GABAergic Dysfunction Participates in the Enhancement of Cortical-BLA Transmission in PRS Mice

**Figure 6.**
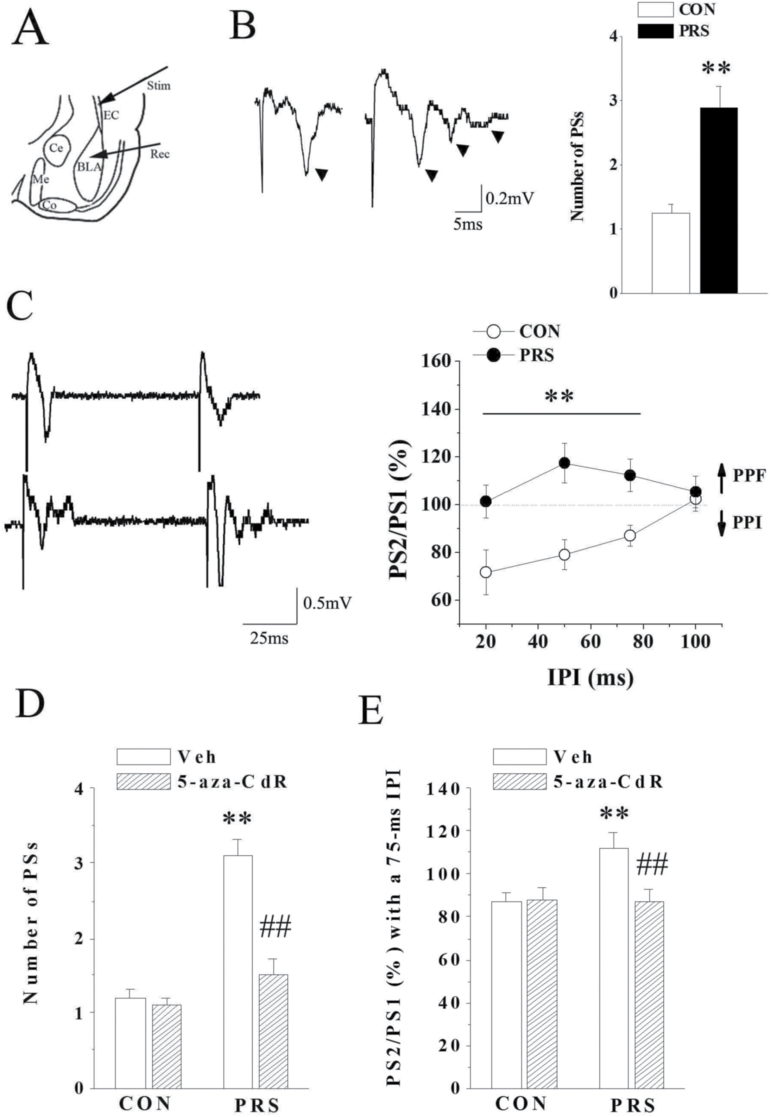
DNA methyltransferase 1 (DNMT1)-mediated GABAergic deficit leads to the potentiation of cortical-basolateral amygdala (BLA) transmission in postnatal stress (PRS) mice. CON and PRS represent control and PRS mice, respectively. (A) Schematic diagram illustrating the placement of stimulating electrode (Stim) and recording electrode (Rec) at cotical-BLA pathway. (B) Left: Basal evoked synaptic response in the BLA of control and PRS mice. PS wave is marked with arrows. Right: The comparison of population spike (PS) number between control and PRS mice. (C) Left: The original results of paired-pulse stimulation with the interval at 50 ms. Right: The comparison of PS2/PS1 with the stimulus interval from 20 to 100 ms between control and PRS mice. Dotted line indicates 100%. ↑ and ↓ represent paired-pulse facilitation (PPF) and paired-pulse inhibition (PPI), respectively. (D and E) Effects of 5-aza-CdR on the number of PSs or the PPI induction in control and PRS mice. All bars represent means±SEM; 2-tailed Student’s *t* test for B and 2-way ANOVA followed by PLSD posthoc test for C, D, and E; **P<.01 between the data of control (n=10) and PRS mice (n=10) or between the data of vehicle-treated control (n=12) and vehicle-treated PRS mice (n=10). ##*P<*.01 between the data of vehicle-treated PRS mice (n=10) and 5-aza-CdR-treated PRS mice (n=10).

GABAergic system in the BLA plays a key role in inhibiting the excitation of the pyramidal cells that receive cortical glutamatergic inputs (Stell et al., 2003; Maguire et al., 2005). Therefore, to evaluate the possibility that epigenetic modification of DNMT1 further changes GABAergic function in the BLA of PRS mice, cortical-BLA glutamate synaptic transmission was detected (Figure 6A). As shown in Figure 6B, in slices from control offspring, a moderate single pulse evoked a PS generated by the synchronous firing of basolateral projecting neurons. By contrast, in slices from PRS mice, the same manner of stimulation evoked the appearance of several additional responses following the main PS in the BLA neurons. The average number of PSs in PRS mice was significantly greater than that for the control group (t_(18)_ = 4.21, *P<*.01). Based on the previous studies (Wang et al., 2001, 2002), the appearance of PSs suggests the possibility that the inhibitory mechanisms that normally restrict repetitive firing in the BLA are impaired in PRS mice. PPI with inter-pulse intervals (IPIs) ranging from 20 to 100 ms were then introduced to determine the inhibitory activity of GABAergic interneuron in the BLA (Isoardi et al., 2004). As shown in Figure 6C, 2-way repeated ANOVA indicated statistically significant effects with PRS (F_(1,20)_=58.024; *P<*.001), IPIs (F_(1,20)_=5.373; *P<*.05), and PRS by IPI interaction (F_(1,20)_=7.916; *P<*.05). PPI was mainly observed with IPIs at 20 to 75 ms in the control mice, whereas paired-pulse facilitation (PPF) instead of PPI was evoked by 20 to 75 ms IPIs in PRS mice (*P<*.01). The PPI-reversed to PPF in PRS mice proves the fact that the decrease of GABAergic inhibitory mechanism results in the increase of the BLA neuronal excitability. Two-way ANOVA displayed main effects of PRS and 5-aza-CdR treatment and their interaction on the number of PSs (PRS: F_(1,42)_=28.62; *P<*.001; 5-aza-CdR: F_(1,42)_=34.35; *P<*.001; PRS×5-aza-CdR: F_(1,42)_=38.01; *P<*.001; Figure 6D) and PS2/PS1 (PRS: F_(1,42)_=5.54; *P<*.05; 5-aza-CdR: F_(1,42)_=4.72; *P<*.05; PRS×5-aza-CdR: F_(1,42)_=4.384; *P<*.05; Figure 6E). The followed PLSD posthoc test further indicated that treatment with 5-aza-CdR abolished the repetitive response (*P<*.01) and recovered PPI (*P<*.01) in the slices from PRS offspring, suggesting the involvement of DNMT1 in the BLA GABAergic deficiency.

### The Intra-BLA Injection of 5-aza-CdR Corrects Anxiety-like Behavior in PRS Mice

**Figure 7.**
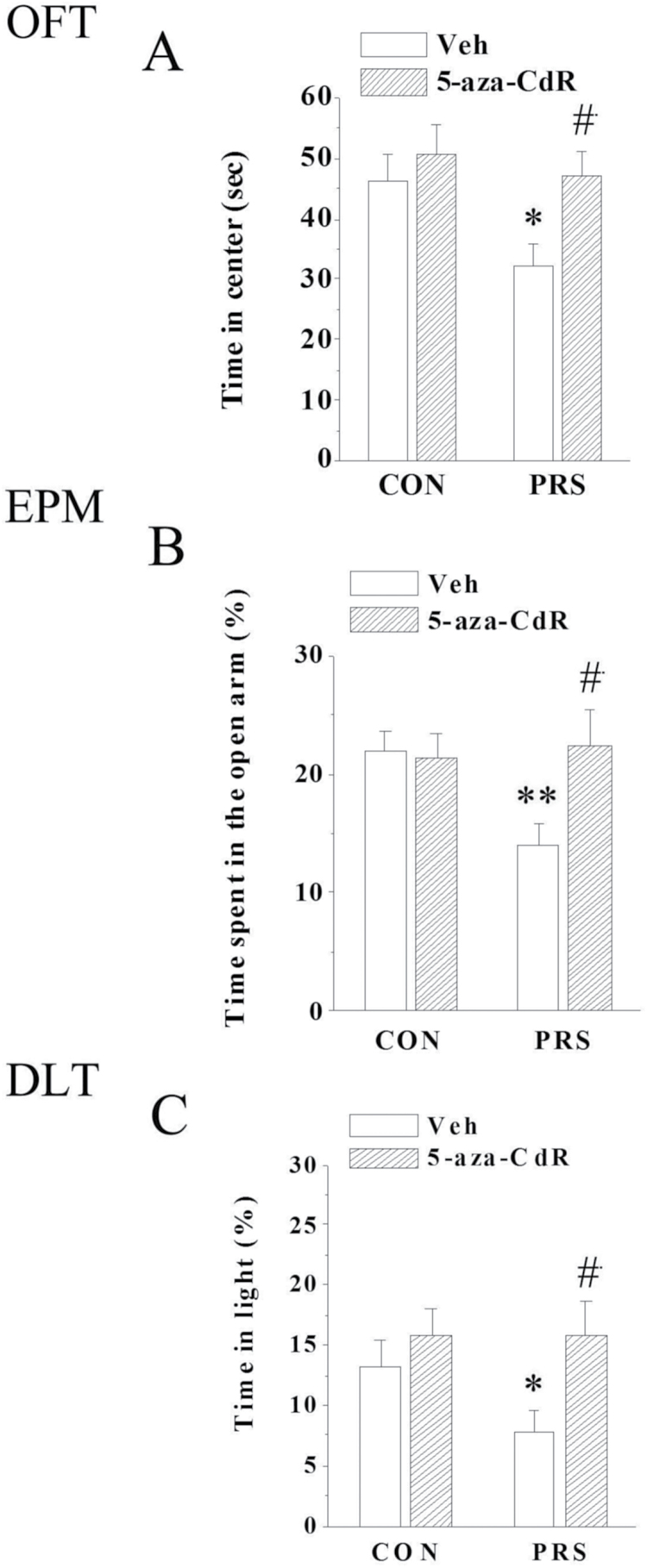
The anxiety-like behaviors in postnatal stress (PRS) mice are corrected by 5-aza-CdR. 5-aza-CdR recovered the shortened center time in the open field test (OFT) (A), improved the decrease in the percentage of time spent in the open arms of the elevated plus maze (EPM) (B), and increased the time in the light box (C) in PRS mice. All values are means±SEM; 2-way ANOVA followed by protected least significant difference (PLSD) posthoc test; *P<.05 and **P<.01 between the data of vehicle-treated control mice (n=14) and vehicle-treated PRS mice (n=14); #*P<*.05 between the data of vehicle-treated PRS mice (n=14) and 5-aza-CdR-treated PRS mice (n=16).

To test whether the behavioral alterations of PRS mice were mediated by epigenetic mechanisms including an increase in DNMT1, an increase of GABAergic promoter methylation, and a downregulation of the GABAergic gene expression, the behavior of PRS mice was evaluated following the repeated microinjection of 5-aza-CdR into the BLA. Two-way ANOVA revealed a main effect of PRS (center time: F_(1,60)_=4.83, *P<*.05; time in the open arm: F_(1,60)_=5.15, *P<*.05; time in light: F_(1,60)_=4.32, *P<*.05) and 5-aza-CdR treatment (center time: F_(1,60)_=5.36, *P<*.05; time in the open arm: F_(1,60)_=5.56, *P<*.05; time in light: F_(1,60)_=5.73, *P<*.05) and a PRS×5-aza-CdR treatment interaction effect (center time: F_(1,60)_=4.95, *P<*.05; time in the open arm: F_(1,60)_=8.64, *P<*.05; time in light: F_(1,60)_=3.02, *P<*.05). The results of the followed PLSD posthoc test further showed that 5-aza-CdR rectified the changed center time in the OFT (*P<*.05; Figure 7A), time spent in the open arm of the EPM (*P<*.05; Figure 7B), and time in the light of the DLT (*P<*.05; Figure 7C), but had no effect in control mice (*P* > 0.05).

## Discussion

This study represents the first demonstration that PRS facilitates anxiety-like behaviors and attenuates GABAergic inhibition in the BLA of female mice offspring, which is at least partly via DNMT1-related epigenetic reprogramming of GABAergic system. In addition, the present data also suggest the long-term neurobehavioral effects of PRS are reversible in the adult period.

It has been widely proved that early life stress causes long-lasting changes in neuroplasticity that result in an increased vulnerability to stress-related disorders in later life (Meaney et al., 2007; Darnaudery and Maccari, 2008; Lupien et al., 2009). The present data that PRS resulted in anxiety-like behaviors and associated endocrinological alterations of adult female mice give further support to the above notion. Female mice exposed to stress in utero represent a new behavioral model of an anxiety-like phenotype, because it recapitulates the potential link between early-life adversity and the pathogenesis of stress-related disorders.

The early development period is crucial for establishing and maintaining epigenetic marks (Reik et al., 2001; Reik, 2007). Epigenetic mechanisms are consequently regarded as the most plausible targets through which early life stress could exert their long-lasting effects. Indeed, accumulating evidence has proved the DNA epigenetic alterations induced by prenatal restrained stress contribute to the complex phenotypes of neuropsychiatric disorders (Matrisciano et al., 2012; Zheng et al., 2016; Papale et al., 2017). DNMTs are important components of the DNA-methylation that dynamically regulates the expression of key molecules involved in brain function. Here, we focused on DNMTs, because these enzymes have been proved to participate in the physiopathology of several neurodevelopmental disorders including anxiety disorder (Ruzicka et al., 2007; Veldic et al., 2007; Guidotti et al., 2011). Our results showed that in these detected enzymes, only the expression of DNMT1 was found to be elevated in the BLA of adult female PRS offspring. To establish in detail whether the altered expression of DNMT1 is expected to result in enrichment of CpG methylation at GAD67 promoters, we then measured levels of 5mC, a CpG methylation marker. As expected, there was significant methylation (high levels of 5mC) found on GAD67 promoters in female PRS mice, and the increased promoter methylation of GAD67 was inversely correlated with the corresponding transcripts. These data suggest PRS results in DNMT1-regulated hypermethylation of GAD67 promotor, thereby inhibiting GAD67 transcription in the BLA. However, we cannot exclude the possibility that histone modifications, another epigenetic regulation of gene transcription, could contribute to the epigenetic modifications detected in female PRS mice.

The GABAergic system in the BLA participates in the regulation of emotional behaviors via inhibiting the excitation of the pyramidal cells that receive cortical glutamatergic inputs (Stell et al., 2003; Maguire et al., 2005). Research in our laboratory and others have established the appearance of multiple PSs in cortical-BLA transmission due to the decreased GABAergic inhibitory effect on the pyramidal neurons (Rodriguez Manzanares et al., 2005). In the present study, the electrophysiological finding that multiple PSs instead of single PS were measured in the BLA slices of female PRS mice strongly suggests that PRS results in the BLA GABAergic deficits. This inference also is supported by the fact that PPI reversed into PPF in the slices of female PRS mice. Guidotti et al. (2005) have found that there is an obvious decrease of GAD67 and other markers of GABAergic interneurons but not neuronal loss in the postmortem brains of patients with neurodevelopmental psychiatric disorders. The present study shows that the overexpression of DNMT1 is responsible for the decrease of GAD67. Hence,it is plausible that DNMT1-induced epigenetic alterations of GABAergic neurons could be the basis for the disturbance of GABA-glutamate neuron interactions in the BLA of female PRS mice.

Traditionally, DNA methylation is thought to be a static process after cellular differentiation. In support of this DNMT stasis hypothesis, the activity and expression of DNMT significantly decreases in differentiated cells and is positively correlated with the proliferative state of cells (Singer-Sam et al., 1990; Yen et al., 1992; Goto et al., 1994). Our present study and others have found that the high level of DNMT1 mRNA is detected in the BLA or hippocampus region of adult mice brain (Levenson et al., 2006; Kadriu et al., 2012). These results suggest a possibility that DNMT stasis is absent in the mature brain. Due to the high expression of DNMT1 in the BLA, it is possible to analyze the role of DNMT1 in long-lasting anxiety using pharmacological methods. In fact, we have found that the local blockade of DNMT1 with 5-aza-CdR in the BLA significantly ameliorated anxiety-like symptoms in PRS mice. It is noteworthy that the same dosage of 5-aza-CdR as that used in PRS mice fails to change anxiety-related behaviors of control mice, suggesting a specificity of action on the epigenetic mechanisms that underlie the behavioral pathology in female PRS mice. Our study indicates PRS leads to the increase of anxiety level in female offspring through epigenetic GABAergic dysfunction. However, another report by Matrisciano et al. (2013) has pointed out the involvement of PRS-induced epigenetic modifications of GABAergic interneurons in schizophrenia in male mice. These data suggest that PRS-induced epigenetic DNA alternations have a sexual dimorphic effect on behavioral outcome. A review by Charil et al. (2010) has pointed out that the sexual dimorphic effect of PRS is related with the sex-difference in brain development. It is therefore inferred that the sex-difference in brain development may be a key factor responsible for sex dimorphisms in neuropsychiatric disorders associated with PRS-induced epigenetic DNA alternations and this possibility requires further investigation.

It has been found that there is a persistent overexpression of DNMT in the brain of PRS mice ranging from birth to adulthood (Matrisciano et al., 2013). We can infer that in female PRS mice, the increase of DNMT1 is probably the result of changes occurring during embryonic life. Further, stress exposure during pregnancy results in the decrease of maternal cares on pups (Pardon et al., 2000). Previous studies suggest that the variations of maternal care have a stable effect on anxiety-mediated behaviors through epigenetic mechanisms (Weaver et al., 2006). Therefore, the change of maternal care could be another factor involved in DNMT1 upregulation in female PRS mice.

In conclusion, these preclinical studies in mice support the concept that the PRS model has construct face validity and pharmacological utility as an experimental epigenetic model of anxiety disorders. We propose that the upregulation of DNMT1 leads to the hypermethylation and increased binding of DNMT1 to GAD67 promoters in PRS model. Furthermore, we propose that drugs that induce promoter hypomethylation and/or DNMT1 downregulation might be useful in correcting anxious behaviors. This means that DNMT1 may represent possible new molecular targets to treat with the long-term neurobehavioral effects of developmental stress exposure.

## Statement of Interest

None.
